# Adult syndromology: challenges, opportunities and perspectives: *Illustrated by the description of four adults with Costello syndrome*

**DOI:** 10.1515/medgen-2024-2023

**Published:** 2024-06-06

**Authors:** Ariane Schmetz, Maria Juliana Ballesta-Martínez, Bertrand Isidor, Ana Berta Sousa, Dagmar Wieczorek, Nuria C. Bramswig

**Affiliations:** Heinrich-Heine-University Dusseldorf Institute of Human Genetics, Medical Faculty and University Hospital Dusseldorf Moorenstr. 5 40225 Dusseldorf Germany; Hospital Clinic University Virgen de la Arrixaca Medical Genetics Section 30120 El Palmar, Murcia Spain; University Hospital Nantes, University of Nantes Medical Genetics Section 8 Quai Moncousu 44007 Nantes France; Santa Maria Hospital / CHULN Pediatrics Department 1649-028 Lisboa Portugal; Heinrich-Heine-University Dusseldorf Institute of Human Genetics, Medical Faculty and University Hospital Dusseldorf Moorenstr. 5 40225 Dusseldorf Germany; Heinrich-Heine-University Dusseldorf Institute of Human Genetics, Medical Faculty and University Hospital Dusseldorf Moorenstr. 5 40225 Dusseldorf Germany

**Keywords:** Adult syndromology, Costello Syndrome, HRAS, intellectual disability, neurodevelopmental disorders

## Abstract

Clinical geneticists and syndromologists have traditionally focused on identifying syndromes in children. However, there is a growing acknowledgment of the need to describe adult phenotypes.

This article provides an overview of the evolving phenotypes of rare genetic syndromes into adulthood, elucidating its challenges, opportunities, and future perspectives. The clinical phenotypes of four adults with Costello syndrome are described to illustrate these aspects.

Phenotypic and genotypic data from four individuals broaden the spectrum of Costello syndrome in adulthood and highlight the high variability in neurocognitive outcome. The clinical data align with previous findings and established genotype-phenotype correlations. Interestingly, two individuals presented with recurrent cancers (bladder cancer and neuroblastoma).

Further studies are imperative to provide reliable information for counselling and management to enable comprehensive understanding of the evolving features of rare syndromic diseases and special health issues into adulthood.

Rare neurodevelopmental genetic disorders often present early in childhood. In consequence, clinical geneticists tend to focus on the description of paediatric populations [1]. These phenotypic descriptions of paediatric cohorts are very useful for the early diagnosis and management in childhood. However, the need for the description of the clinical course into adulthood and in adults becomes more and more evident. On the one hand, the diagnosed individuals are getting older requiring management guidelines for adult individuals. On the other hand, some of the parents’ / caregivers’ concerns revolve around their child’s future as an adult. Common questions include whether the affected child will need assistance in its daily adult life and whether there is an increased risk for other diseases in adulthood. For most monogenic neurodevelopmental disorders, this information about the adult phenotype is limited or lacking in the scientific literature.

## Overview of adult syndromology

Clinical geneticists and syndromologists have focused predominantly on identifying syndromes in children, driven by the therapeutic imperative of early diagnosis and its implications for the parents’ future family planning. Salomon and Salomon investigated the distribution of children and adult photographs within the renowned textbook “Smith’s Recognizable Patterns of Human Malformation, 8th Edition (2021)” [1]. In the paediatric age group they found 1188 photographs (87 %) compared to 85 photographs in the adult age group (6 %). In October 2023, we analysed the number of publications describing paediatric and adult individuals with genetically confirmed Coffin-Siris syndrome using the following setup (adapted from [2]). The first Coffin-Siris syndrome-related gene was described in 2012 [3]. To include mainly genetically confirmed individuals we searched the scientific database PubMed for “Coffin-Siris syndrome” and limited the results to publications from 2012 onward. This search yielded 221 results. Further narrowing the aforementioned query to publications specifically involving adult patients with Coffin-Siris syndrome (Filter: “Age: Adult: 19+ years”) resulted in 27 matches. A thorough review of these 27 publications revealed that 12 did not include adult patients with Coffin-Siris syndrome. Among the remaining 15 relevant results, seven were publications from predominantly paediatric cohorts with few adult cases, three were publications showing vertical transmission of a pathogenic variant, three were single-case descriptions, and two were predominantly paediatric cohorts with a range extending into adulthood, but with an unknown age structure [2]. In these 15 publications, a total of 25 adult cases were identified some including no or only minimal clinical details. In contrast to this small number of 25 adult individuals, one single cohort with up to 208 mainly paediatric Coffin-Siris syndrome individuals has been published [4].

Using the same analysis in December 2023 the ratios between all publications vs. publications including adult patients (19+ years) was 61 to 7 for Nicolaides-Baraitser syndrome, 427 to 86 for Costello syndrome, 113 to 23 for Cardiofaciocutaneous syndrome and 171 to 20 for Pitt-Hopkins syndrome [5]. Given this rough analysis there are at least 5–9 times more publications including children than adults. However, in most publications including adult individuals, the majority of described individuals are children and paediatric cohorts tend to be larger. Therefore, in clinical practice, the difference in available clinical information appears even more pronounced resulting in difficulties concerning counselling, care, and treatment of adult individuals with rare genetic syndromes.

Taylor and colleagues focused on transition and adult genetics and hereby divided the adult population into two groups [6]: First, the “walking diagnosable”, describing adults that have an undiagnosed, potentially recognizable genetic condition; and second the “walking diagnosed”, describing adults that already received their genetic diagnosis.

To address and improve healthcare for the first group of “walking diagnosable” individuals broader access to and information on genetic testing is needed. To apply a widened testing strategy, primary health care providers would have to be sensitized to the possible occurrence of rare genetic syndromes in their adult patients’ population. In Germany, the government has implemented the *Medizinische Zentren für Menschen mit Behinderungen (MZEB)* to assure transition and continuity of care of multimorbid children into adulthood and healthcare adapted to their special needs. For more in-depth details on MZEB, please refer to Article **“MZEB (Medizinisches Zentrum für Erwachsene mit Behinderungen) Bedburg-Hau – Genetic testing in adults with mental disabilities and developmental and psychiatric disorders”** of the present issue. In these centres, many “walking diagnosable” individuals are gathered. Others and we have started to offer genetic testing in and support to these centres. These genetic (re-)evaluations of adults with intellectual disability enable the establishment of genetic diagnosis at a high percentage and often has direct impact on clinical management optimization [7].

The needs of the second group of “walking diagnosed” individuals is broader knowledge of the clinical phenotype and evolving features of the syndrome into adulthood. To investigate the current situation in adult phenotypes in syndromology, we did an extensive but not exhaustive literature search. This search revealed some early publications of larger adult cohorts. In the following we highlight some findings. In 1987, Greenswag published a description on 232 adults with Prader-Willi syndrome [8]. In 1990, Dr. Udwin published a survey based on questionnaires completed by caregivers about 119 adults with Williams syndrome [9]. However, for both syndromes the molecular basis was not completely understood at that time raising the question whether all individuals received the correct diagnosis. Marcelis and colleagues described four adult individuals with Wolf-Hirschhorn syndrome in 2001 [10]. In 2002, Hunter published an update of the clinical history of their previously reported patients with Coffin-Lowry syndrome after 20 years [11]. Both authors highlighted the need for more information about long-term outcomes and follow-ups [10,11]. Binder and colleagues published a survey about 45 adult individuals with Noonan syndrome [12] and Douzgou and colleagues published a families-reported questionnaire-based study on the natural history of 87 adults with Rubinstein-Taybi syndrome [13]. Recently, our group published the first cohort of 35 adult individuals with Coffin-Siris syndrome [14]. We were able to document interesting features in adults with Coffin-Siris syndrome such as obesity and behavioural anomalies.

For other more frequent neurodevelopmental disorders like the 22q11.2 deletion syndrome, an interdisciplinary team of professionals has developed practical management guidelines for affected adult individuals [15]. Likewise, the clinical guideline for the management of Noonan Syndrome developed by the Noonan Syndrome Guideline Development Group (DYSCERNE) and available at the website rasopathiesnet.org includes information about management in adulthood [16], as well as the guidelines on the management of Phelan-McDermid syndrome [17] and Alström syndrome [18].

To enhance genetic counselling for all individuals with rare syndromes and their caregivers, we initiated a study on the evolving phenotypes of rare genetic syndromes. In the following, we will describe four adult individuals with Costello syndrome.

**Table 1:** Heterozygous pathogenic variants in *HRAS* (NM_005343) in the four participants

	Individual 1	Individual 2	Individual 3	Individual 4
Variant in cDNA	c.35G>C	c.34G>A	c.34G>T	c.37G>T
Predicted protein change	p.(Gly12Ala)	p.(Gly12Ser)	p.(Gly12Cys)	p.(Gly13Cys)
Genomic position [GRCh38/hg38]	11: 534288	11: 534289	11: 534289	11: 534286
Origin of the variant	de novo	de novo	de novo	both parents not tested

## Methods

We performed a data collection using questionnaires through a collaborative effort. Detailed methodology has been described elsewhere [2,14].

Patients were eligible to participate in this study at the age of 18 years or older with a genetically confirmed diagnosis of Costello syndrome. If the individuals had been part of a previous publication, it needed to be cited.

## Costello syndrome (CS)

Dr. Costello described this new syndrome with “mental subnormality and nasal papillomata” in 1977 [19]. In 2005, Aoki and colleagues identified heterozygous pathogenic or likely pathogenic variants in *HRAS* as the genetic basis of the syndrome [20]. Most *HRAS* variants are highly recurrent and some genotype-phenotype correlations have been reported [21]. In most individuals with CS the causal variant involves the amino acid glycine in position 12, with Gly12Ser being the most prevalent substitution reported in 60–80 % CS-individuals [22,23].

CS presents with characteristic features affecting multiple organ systems. In general, the phenotypic spectrum of CS is broad. Common manifestations include failure to thrive in infancy due to severe postnatal feeding difficulties, short stature, macrocephaly or relative macrocephaly, developmental delay, and intellectual disability. Facial features are distinctive, marked by full lips, a large mouth, full nasal tip, and coarse features. Other notable traits encompass curly and often sparse, fine hair, loose skin with deep palmar and plantar creases, and papillomata in facial and perianal regions. Many CS-affected individuals show hypotonia, orthopaedic issues and cardiac involvement [19–24]. Individuals with CS have an increased cancer risk. In a recent systematic review [23] 9.2 % of the included individuals with CS had cancer and specific standardised incidence ratios for each type of cancer have been calculated.

In the following, we describe phenotypic and genotypic data from four individuals, three females and one male with Costello syndrome. The mean age at data collection was 23.7 years, with a range from 20.2 to 27.1. Individual 4 had been previously reported at the age of 6 years and 8 months (Gripp et al. 2011: Ind CS-PT1[25]).

Individual 1, 2, and 3 had *de novo* variants in amino acid position 12 of HRAS: Gly12Ala, Gly12Ser, and Gly12Cys, respectively. Individual 4 harboured the well-known HRAS variant Gly13Cys, parental testing had not been performed.

In the following we will describe the clinical findings in the four individuals with genetically confirmed CS. A summary of the clinical results is shown in Table 2.

### The four CS individuals presented with polyhydramnios during pregnancy, feeding difficulties, short stature, and (relative) macrocephaly

Short stature was a constant feature in this small group of individuals (adult height ranging from 131.5 cm to 150 cm). Individual 1 was diagnosed with growth hormone insufficiency, treated with growth hormone and reached an adult height of 145.5 cm. Three individuals had macrocephaly. Individual 3 displayed relative macrocephaly with OFC at –2.52 SD and height and weight at <–8 SD. Two individuals had normal adult weight (BMI 24.6 and 23.6 kg / m^2^), one individual was underweight (BMI 15.9 kg / m^2^). For individual 3 height and weight was not available at the same time point therefore BMI calculation was not possible. All individuals had reported feeding difficulties in infancy with 3/4 requiring tube feeding. Another constant feature in this group was polyhydramnios during pregnancy (4/4).

### All individuals present clinical phenotypes related to joint contractures

Individual 3 developed severe contractures during his first years of life leading to hip and knee tenotomies at 13 years of age. He showed motor regression in relation to talipes at the age of 13 years and focal epilepsy at 17 years. Individual 1 also had congenital talipes, which were surgically corrected and developed scoliosis during adolescence. Individuals 2, 3, and 4 had movement restrictions due to congenital and acquired contractures of joints.

**Figure 1:**
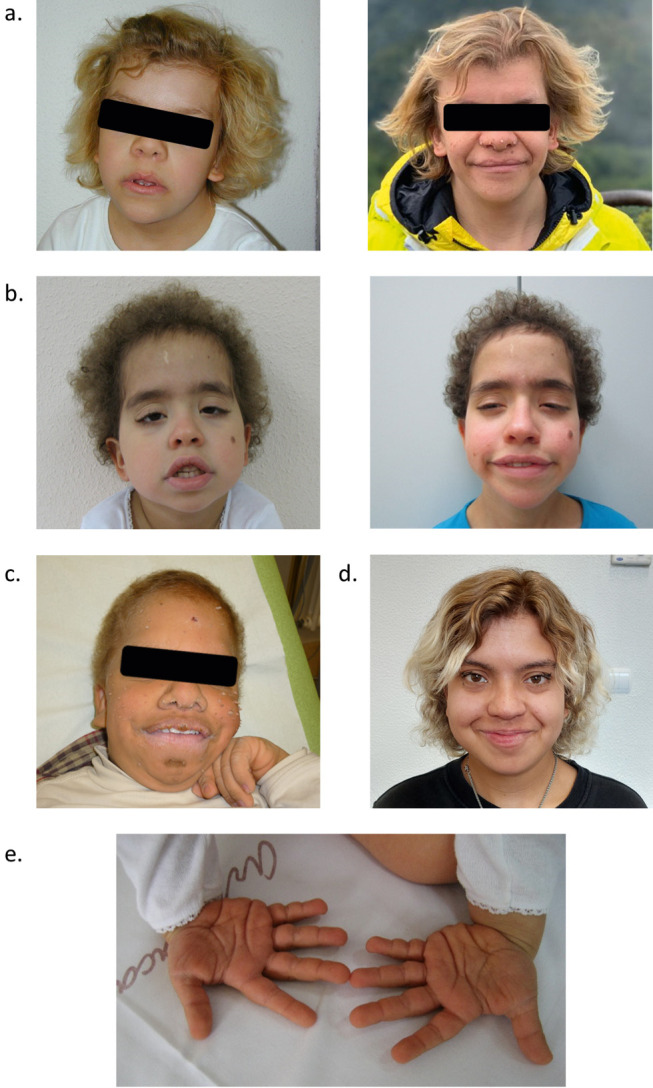
CS-related phenotype. a Individual 1 in childhood (left) and adulthood (27 years of age, right). b. Individual 2 at five (left) and eleven (right) years of age. c. Individual 3 (13 years of age) d. Individual 4 (20 years of age). e. Palmar view of hands with typical loose skin with deep palmar creases (individual 2, 5 years of age).

### Recurrent health problems in four adult CS individuals

One individual had a supravalvular aortic stenosis at birth and needed beta blockers until the age of 14 years (individual 1). Another individual had congenital hypertrophic cardiomyopathy requiring beta blockers (individual 3). Individual 2 had obstructive hypertrophic cardiomyopathy with aortic and mitral valve insufficiency diagnosed at the age of 5 years requiring cardiac surgery for hypertrophy, aortic valve replacement and mitral valvuloplasty at the age of 21 years.

Three out of four individuals had congenital nystagmus. Additional eye conditions included myopia, exotropion, as well as retinal and optic pathways abnormalities. No one displayed impaired hearing.

At the age of 17 years individual 3, who is not able to walk, had a Z-score of –6SD in bone density test indicating osteoporosis.

### Half of the CS individuals presented with recurrent (malignant) tumors

Individual 1 (HRAS Gly12Ala) developed bladder cancer at the age of 11, 14, and 18 years. She also presented with recurrent intraductal papillomata of the breast. Individual 3 developed neuroblastoma at the age of 1 and 6 years. The second adrenal gland was surgically removed due to intense FDG uptake in PET-CT; however, neuroblastoma could not be confirmed upon histopathological examination.

Both individual 1 and individual 3 developed papillomata of the face and of the face and limbs in childhood, respectively. In individuals 2 and 4 no papillomata were reported.

### The neurocognitive outcome was highly variable

All participants had developmental delay with individual 3 not achieving independent walking and staying non-verbal. Individuals 1, 2, and 4 were verbal and able to walk. The cognitive outcomes ranged from severe intellectual disability (individual 3) to normal intellect for individual 4, who is currently attending university and able to live independently. Individual 1 is working in a sheltered workshop.

For two out of four individuals (individual 1 and individual 3) behavioural anomalies were reported. Both present with stereotypic movements. In addition, individual 1 was described to be sensitive to noises and to have a friendly personality and high empathy.

### Facial features of CS are consistent

All individuals displayed the typical facial features with broad coarse face, curly and sometimes sparse hair, short neck, thick lips, bulbous nose, and smooth philtrum (Figure 1). The characteristic deep palmar creases were reported in three individuals and the facial papillomata in two individuals.

**Table 2:** Clinical characteristics of presented adults with Costello syndrome

**Clinical characteristics**	**Costello syndrome**
**Growth parameters**	
Short stature	4/4
Underweight	1/3
Overweight	0/3
Microcephaly	0/4*^a^*
Macrocephaly	3/4
Relative Macrocephaly	1/4
Polyhydramnios during pregnancy	4/4
Primary macrocephaly	1/3
**Health issues by system**	
Brain anomalies	1/4
Seizures	1/4
Cardiovascular anomalies	3/4
Respiratory anomalies	1/4
Feeding difficulties in infancy	4/4
Tube feeding	3/4
Constipation	0/4
Gastrointestinal anomalies	1/4
Kidney anomalies	1/4
Urogenital anomalies	0/4
Endocrine anomalies	2/4
Delayed puberty	2/4
Dermal anomalies	3/4
Papillomata	2/4
Callous	1/4
Curly hair	4/4
Eye conditions	4/4
Hearing impairment	0/4
Hematological anomalies	0/4
Recurrent infections	1/4
Malignancies	2/4
Musculoskeletal anomalies	4/4
contractures	4/4
**Cognitive Outcome**	
Intellectual disability	3/4
Severity of ID: none (IQ>70) borderline (IQ<70) very mild (IQ<65) mild (IQ<50) moderate (IQ<35) severe (IQ<20)	1/4 1/4 1/4 1/4
**Developmental outcome**	
Delayed age of sitting*^b^*	2/2
Delayed age of walking*^b^*	4/4
Delayed age of speaking*^b^*	4/4
Currently non-ambulatory	1/4
Currently non-verbal	1/4
Currently able to tell stories	3/4
In need of help for daily hygiene	1/4
In need of help for eating	1/4
Able to live independently	1/4
Occupation	2/4
**Behavioral anomalies**	2/4
Stereotypic movements	2/4

*^a^* With an OFC below –2SD but height and weight below –8SD, individual 3 has relative macrocephaly and was not listed as microcephalic.

*^b^* Delayed age of sitting: later than 8 months, walking: later than 18 months and speaking: later than 15 months (>75 % percentile following Zubler et al. [26])

### Discussion

With this study, we add detailed phenotype and genotype descriptions of four adult individuals with CS to the current scientific literature and further highlight the broad spectrum of this syndrome in adulthood. The clinical data of the four presented individuals is consistent with previously published data and the established genotype-phenotype correlations [21].

Individual 1 with *HRAS* Gly12Ala had recurrent bladder carcinomata. A higher rate of malignancies has been established for those individuals [23]. In their 2019 review, Gripp and colleagues cited transitional cell carcinoma of the bladder as the only malignancy reported in adults with CS, based on reports by Beukers and colleagues and White and colleagues [21,27,28]. In 2022, Leoni and colleagues performed a study about the prevalence of bladder cancer in CS [29]. They identified premalignant and malignant bladder lesions in eleven out of the 13 mostly adult individuals with CS. Moreover, individual 1 presented with recurrent and multifocal intraductal papillomata of the breast. Intraductal papillomata of the breast had previously been reported by White and colleagues in 2 out of 17 individuals with CS and in one Spanish case report [28,30].

Individual 2 with *HRAS* Gly12Ser presents with classical features of CS and had no history of malignancy at the age of 22 years. This variant represents the most common variant in individuals with a molecular diagnosis of CS and is associated with a lower rate of cancer [23].

Individual 3 with *HRAS* Gly12Cys presents with a severe phenotype. However, no lung abnormalities and pleural or cardiac effusion, that are commonly associated with this variant [21], are known for the present individual but he presented neonatal respiratory distress. He had developed neuroblastoma at one and 6 years of age. The diagnosis of CS was established by direct *HRAS* sequencing in the pre-exome-sequencing era. In view of his severe phenotype, it could be argued that his phenotype could be partially explained by a second diagnosis rather than represent the severe end of the CS spectrum. In such cases, a genetic re-evaluation at the current state of the art could be considered.

Individual 4 with *HRAS* Gly13Cys presents with milder symptoms, both on the somatic and neurocognitive side, she has had some degree of developmental delay in childhood but now attends university. Gripp and colleagues described a cohort of 12 Gly13Cys individuals, including individual 4 (Ind CS-PT1), with some other individuals having low-normal IQ testing results [25]. There are rare reports of vertical transmission of *HRAS* variants in the scientific literature [31,32]. In the above-mentioned cohort of 12 individuals the authors compared phenotypic frequencies to individuals with the Gly12Ser variant. These individuals with Gly13Cys presented with significantly less short stature without use of growth hormone, atrial tachycardia, ulnar deviations, papillomata, and ulnar deviations of the wrist. In this cohort, the absence of malignancy did not reach significance. However, in their review about cancer in CS, Astiazaran-Symonds and colleagues did not identify any individuals with the Gly13Cys variant and an associated malignancy [23].

In 2020, Shikany and colleagues published a cohort of 20 adolescents and adults with CS (age range 16 to 40 years) [33]. In their cohort, they identified a high prevalence of anxiety, which was not documented in our CS individuals. They identified the need of monitoring for progressive contractures. In their cohort 65 % of CS individuals needed walking assistance [33]. In this study, all individuals had some kind of joint contractures but only one individual was not able to walk independently. Lastly, they highlighted the fact that from their cohort of 20 individuals with CS, nobody has had a malignancy in adulthood. The mutational spectrum included in their work was *HRAS* Gly12Ser and Gly13Cys / Gly13Asp. In the recent review of cancer in CS, individuals with the Gly12Ser variant were found to have lower cancer risk [23]. Moreover, they identified no individuals with cancer harbouring the variants Gly13Cys / Gly13Asp in the scientific literature. Therefore, the low incidence of cancers in their cohort could be linked to the mutational spectrum. In this study, individual 1 (Gly12Ala) and individual 3 (Gly12Cys) presented with recurrent malignancies from childhood into adulthood and in childhood-only, respectively. Rhadomyosarcoma, bladder cancer, and neuroblastoma are the most common cancers in individuals with CS [23].

Fortunately, management guidelines for CS individuals exist. Gripp and colleagues highlighted the need of further information about specific concerns in adulthood of CS individuals [21]. In their 2019 guidelines, they briefly touch on the challenges experienced in adulthood and the necessary management strategies. Shikany and colleagues provided some management advice based on their description of 20 adult CS individuals [33]. In addition, Leoni and colleagues dedicated a whole section to adults in their work on the current perspectives of multidisciplinary management of CS [24]. They highlighted the importance of monitoring bone homeostasis. One out of four individuals from the present report was diagnosed with osteoporosis before adulthood. The bone mineralisation status was not available for the other three individuals.

The four individuals described in this study highlight the high variability in neurocognitive outcome, with one individual not being able the speak (individual 3) and another one currently attending university (individual 4). Two of the described individuals presented with recurrent cancers, which to the best of our knowledge is an uncommon feature.

## Conclusions

In the scientific community, there is rising awareness about the lack of phenotypic and genotypic information in adult syndromology and the associated difficulties in counselling and care of these individuals. Others and we have started to fill this knowledge gap (1) by collecting clinical information of homogeneous adult cohorts of individuals with different rare syndromes and (2) by making genetic testing and re-evaluations more accessible to adults with neurocognitive disorders. These studies have yielded important information for healthcare providers and families with young children and adults with these rare syndromes. Further studies with even larger adult cohorts are indispensable in order to provide reliable information for proper counselling and management. These studies need (1) the support of the entire genetic community to collect sufficiently large data sets and (2) improved multidisciplinary management of adult individuals with neurocognitive disorders including the possibility of genetic testing.

## References

[1] Solomon A. M., Solomon B. D. (2023). Age-related survey of clinical genetics literature and related resources. Am. J. Med. Genet. C Semin. Med. Genet.

[2] Schmetz A. (2023). Entwicklung des Phänotyps vom Kind zum Erwachsenen bei Menschen mit seltenen Syndromen: das Coffin-Siris-Syndrom. Entwicklung des Phänotyps vom Kind zum Erwachsenen bei Menschen mit seltenen Syndromen: das Coffin-Siris-Syndrom.

[3] Hoyer J., Ekici A. B., Endele S., Popp B., Zweier C., Wiesener A., Wohlleber E., Dufke A., Rossier E., Petsch C. (2012). Haploinsufficiency of ARID1B, a member of the SWI / SNF-a chromatin-remodeling complex, is a frequent cause of intellectual disability. Am. J. Hum. Genet.

[4] Vasko A., Drivas T. G., Schrier Vergano S. A. (2021). Genotype-Phenotype Correlations in 208 Individuals with Coffin-Siris Syndrome. Genes (Basel).

[5] (2004). Bethesda (MD). Bethesda (MD).

[6] Taylor M. R., Edwards J. G., Ku L. (2006). Lost in transition: challenges in the expanding field of adult genetics. Am. J. Med. Genet. C Semin. Med. Genet.

[7] Knopp C., Steiner R., Lausberg E., Hoegen C. V., Busse S., Meyer R., Eggermann K., Schuler H., Begemann M., Eggermann T. (2022). Genetic (Re-)evaluation to Optimize the Care of Adults With Intellectual Disability. Dtsch Arztebl Int.

[8] Greenswag L. R. (1987). Adults with Prader-Willi syndrome: a survey of 232 cases. Dev. Med. Child Neurol.

[9] Udwin O. (1990). A survey of adults with Williams syndrome and idiopathic infantile hypercalcaemia. Dev. Med. Child Neurol.

[10] Marcelis C., Schrander-Stumpel C., Engelen J., Schoonbrood-Lenssen A., Willemse A., Beemer F., Sigaudy S., Missirian C., Philip N., Fryns J. P. (2001). Wolf-Hirschhorn (4P-) syndrome in adults. Genet. Couns.

[11] Hunter A. G. (2002). Coffin-Lowry syndrome: a 20-year follow-up and review of long-term outcomes. Am. J. Med. Genet.

[12] Binder G., Grathwol S., von Loeper K., Blumenstock G., Kaulitz R., Freiberg C., Webel M., Lissewski C., Zenker M., Paul T. (2012). Health and quality of life in adults with Noonan syndrome. J. Pediatr.

[13] Douzgou S., Dell’Oro J., Fonseca C. R., Rei A., Mullins J., Jusiewicz I., Huisman S., Simpson B. N., Vyshka K., Milani D. (2022). The natural history of adults with Rubinstein-Taybi syndrome: a families-reported experience. Eur. J. Hum. Genet.

[14] Schmetz A., Ludecke H. J., Surowy H., Sivalingam S., Bruel A. L., Caumes R., Charles P., Chatron N., Chrzanowska K., Codina-Sola M. (2024). et al. Delineation of the adult phenotype of Coffin-Siris syndrome in 35 individuals. Hum. Genet.

[15] Fung W. L., Butcher N. J., Costain G., Andrade D. M., Boot E., Chow E. W., Chung B., Cytrynbaum C., Faghfoury H., Fishman L. (2015). Practical guidelines for managing adults with 22q11.2 deletion syndrome. Genet. Med.

[16] (2011). Management of Noonan Syndrome. Management of Noonan Syndrome.

[17] Srivastava S., Sahin M., Buxbaum J. D., Berry-Kravis E., Soorya L. V., Thurm A., Bernstein J. A., Asante-Otoo A., Bennett W. E., Jr., Betancur C. (2023). Updated consensus guidelines on the management of Phelan-McDermid syndrome. Am. J. Med. Genet. A.

[18] Tahani N., Maffei P., Dollfus H., Paisey R., Valverde D., Milan G., Han J. C., Favaretto F., Madathil S. C., Dawson C. (2020). Consensus clinical management guidelines for Alstrom syndrome. Orphanet J. Rare Dis.

[19] Costello J. M. (1977). A new syndrome: mental subnormality and nasal papillomata. Aust. Paediatr. J.

[20] Aoki Y., Niihori T., Kawame H., Kurosawa K., Ohashi H., Tanaka Y., Filocamo M., Kato K., Suzuki Y., Kure S. (2005). Germline mutations in HRAS proto-oncogene cause Costello syndrome. Nat. Genet.

[21] Gripp K. W., Morse L. A., Axelrad M., Chatfield K. C., Chidekel A., Dobyns W., Doyle D., Kerr B., Lin A. E., Schwartz D. D. (2019). Costello syndrome: Clinical phenotype, genotype, and management guidelines. Am. J. Med. Genet. A.

[22] Gripp K. W., Lin A. E. (2012). Costello syndrome: a Ras / mitogen activated protein kinase pathway syndrome (rasopathy) resulting from HRAS germline mutations. Genet. Med.

[23] Astiazaran-Symonds E., Ney G. M., Higgs C., Oba L., Srivastava R., Livinski A. A., Rosenberg P. S., Stewart D. R. (2023). Cancer in Costello syndrome: a systematic review and meta-analysis. Br. J. Cancer.

[24] Leoni C., Viscogliosi G., Tartaglia M., Aoki Y., Zampino G. (2022). Multi-disciplinary Management of Costello Syndrome: Current Perspectives. J Multidiscip Healthc.

[25] Gripp K. W., Hopkins E., Sol-Church K., Stabley D. L., Axelrad M. E., Doyle D., Dobyns W. B., Hudson C., Johnson J., Tenconi R. (2011). et al. Phenotypic analysis of individuals with Costello syndrome due to HRAS p.G13C. Am. J. Med. Genet. A.

[26] Zubler J. M., Wiggins L. D., Macias M. M., Whitaker T. M., Shaw J. S., Squires J. K., Pajek J. A., Wolf R. B., Slaughter K. S., Broughton A. S. (2022). Evidence-Informed Milestones for Developmental Surveillance Tools. Pediatrics.

[27] Beukers W., Hercegovac A., Zwarthoff E. C. (2014). HRAS mutations in bladder cancer at an early age and the possible association with the Costello Syndrome. Eur. J. Hum. Genet.

[28] White S. M., Graham J. M., Jr, Kerr B., Gripp K., Weksberg R., Cytrynbaum C., Reeder J. L., Stewart F. J., Edwards M., Wilson M. (2005). The adult phenotype in Costello syndrome. Am. J. Med. Genet. A.

[29] Leoni C., Paradiso F. V., Foschi N., Tedesco M., Pierconti F., Silvaroli S., Diego M. D., Birritella L., Pantaleoni F., Rendeli C. (2022). Prevalence of bladder cancer in Costello syndrome: New insights to drive clinical decision-making. Clin. Genet.

[30] Arpa E., Domínguez-Cunchillos F., Martínez-Montero I., De Miguel C., Moras N. (2007). [Intraductal breast papillomas in patients with Costello syndrome]. Cir Esp.

[31] Gripp K. W., Hopkins E., Serrano A., Leonard N. J., Stabley D. L., Sol-Church K. (2012). Transmission of the rare HRAS mutation (c. 173C > T; p.T58I) further illustrates its attenuated phenotype. Am. J. Med. Genet. A.

[32] Gripp K. W., Sol-Church K., Smpokou P., Graham G. E., Stevenson D. A., Hanson H., Viskochil D. H., Baker L. C., Russo B., Gardner N. (2015). An attenuated phenotype of Costello syndrome in three unrelated individuals with a HRAS c.179G>A (p.Gly60Asp) mutation correlates with uncommon functional consequences. Am. J. Med. Genet. A.

[33] Shikany A. R., Baker L., Stabley D. L., Robbins K., Doyle D., Gripp K. W., Weaver K. N. (2020). Medically actionable comorbidities in adults with Costello syndrome. Am. J. Med. Genet. A.

